# Supplementary material for: Diverse Genetic Landscape of Suspected Retinitis Pigmentosa in a Large Korean Cohort
Source: Genes (Basel). 2021 Apr 30;12(5):675. doi: 10.3390/genes12050675 (PMC8146864; doi:10.3390/genes12050675)

Table S1. Targeted next-generation sequencing panel consisted of 88 genes associated with RP.

ABCA4	*AIPL1*	*ALMS1*	*ARL6*	*ATF6*	*BBS2*	*BEST1*	*C2orf71*	*CA4*	*CACNA1F*
CACNA2D4	*CDH23*	*CDHR1*	*CERKL*	*CLRN1*	*CNGA1*	*CNGB1*	*CNNM4*	*CRB1*	*CRX*
DFNB31	*DHDDS*	*EYS*	*FAM161A*	*FLVCR1*	*FSCN2*	*GUCA1A*	*GUCA1B*	*GUCY2D*	*HARS*
HGSNAT	*IDH3B*	*IFT140*	*IFT172*	*IL1A*	*IL1B*	*IMPDH1*	*IMPG2*	*KCNV2*	*KLHL7*
LRAT	*MAK*	*MFRP*	*MYO7A*	*NPHP4*	*NR2E3*	*NRL*	*OFD1*	*PANK2*	*PCDH15*
PDE6A	*PDE6B*	*PDE6C*	*PDE6G*	*PDZD7*	*PITPNM3*	*PROM1*	*PRPF3*	*PRPF31*	*PRPF6,*
PRPF8	*PRPH2*	*RBP3*	*RDH12*	*RGR*	*RHO*	*RIMS1*	*RLBP1*	*ROM1*	*RP1*
RP2	*RPE65*	*RPGR*	*RPGRIP1*	*RRM2B,*	*SAG*	*SEMA4A*	*SNRNP200*	*SPATA7*	*TOPORS*
TTC8	*TTPA*	*TULP1*	*UNC119*	*USH1G*	*USH2A*	*ZNF408*	*ZNF513*		

Table S2. The proportion of pathogenic variants according to segregation analysis.

	Initial analysis	After re-assessment		
		With familial segregation test	Without familial segregation test	Total
No. of variants, n	161	58	129	181
PV, n (%)[a]	45 (28.0)	18 (31.0)	31 (24.0)	47 (26.0)
LPV, n (%)[a]	55 (34.2)	13 (22.4)	48 (37.2)	58 (32.0)
VUS, n (%)[a]	61 (37.9)	27 (46.6)	50 (38.8)	76 (42.0)

Abbreviations: PV, pathogenic variant; LPV, likely pathogenic variant; VUS, variant of uncertain significance

[a] The percentages were calculated for a subgroup according to segregation tests.

Table S3. Clinical characteristics according to the presence of family history.

		With family history n = 91 (32.6%)	Without family history n = 188 (67.4%)	*P value*
Sex, M:F, n (%)		42:49 (46.2:53.8)	89:99 (47.3:52.7)	0.852[a]
Age at genetic examination, years		50.7±15.5	46.1±15.7	0.655[b]
Age at symptom onset, years		23.2±15.5	26.8±17.7	0.011[b]
Age at diagnosis, years		41.9±15.7	40.6±15.2	0.383[b]
BCVA, LogMAR	RE	1.1±1.2	0.6±0.9	0.000[b]
	LE	1.0±1.1	0.7±0.9	0.000[b]
No. of probands with detected variants, n (%)		66 (72.5)	105 (55.9)	0.007[a]

Abbreviations: M, male; F, female; SD, standard deviation; BCVA, best-corrected visual acuity; LogMAR, logarithm of the minimum angle of resolution; RE, right eye; LE, left eye

[a] Pearson chi square test

[b] Independent t test was used and *p*-values < 0.05 were considered statistically significant.

Table S4. A list of variants detected in targeted next-generation sequencing and whole exom sequencing in this study.

Gene	NM number	HGVS DNA change	HGVS protein change	Zygosity	chromosome	inheritance	ACMG criteria	
ABCA4	NM_000350.2	c.1699G>A	p.Val567Met	hetero	1	AD/AR	LPV	
ABCA4	NM_000350.2	c.1760+2T>G		hetero	1	AD/AR	PV	
ABCA4	NM_000350.2	c.1933G>A	p.Asp645Asn	hetero	1	AD/AR	VUS	
ABCA4	NM_000350.2	c.5063dup	p.Ser1689LeufsTer98	hetero	1		LPV	Novel
ABCA4	NM_000350.2	c.5881G>A	p.Gly1961Arg	hetero	1	AD/AR	LPV	
ABCA4	NM_000350.2	c.880C>T	p.Gln294Ter	hetero	1	AD/AR	PV	
ABCC6	NM_001171.5	c.3703C>T	p.Arg1235Trp	hetero	16	AD	PV	
ABCC6	NM_001171.5	c.3698T>C	p.Val1233Ala	hetero	16	AD/AR	VUS	
ADGRV1	NM_032119.3	c.15608A>C	p.Glu5203Ala	hetero	5	AR/DR	VUS	
ADGRV1	NM_032119.3	c.6221T>C	p.Val2074Ala	hetero	5	AR/DR	VUS	
ADGRV1	NM_032119.3	c.7071_7073G		hetero	5	AR	LPV	Novel
ADGRV1	NM_032119.3	c.7406G>A	p.Trp2469Ter	hetero	5	AR	PV	
ARL6	NM_001278293.2	c.281T>C	p.Ile94Thr	homo	3	AR/DR	PV	
BEST1	NM_001139443.1	c.19_21C		hetero	11		LPV	
BEST1	NM_001139443.1	c.857C>A	p.Pro286His	hetero	11		VUS	
BEST1	NM_001300786.1	c.908T>C	p.Ile303Thr	hetero	11	AD/AR	VUS	
BEST1	NM_001139443.1	c.989T>C	p.Ile330Thr	hetero	11	AD	VUS	
C21orf2	NM_001271440.1	c.319T>C	p.Tyr107His	homo	21	AR	LPV	
CAPN5	NM_004055.4	c.976A>T	p.Tyr326Ser	hetero	11	AD	VUS	Novel
CDH23	NM_022124.5	c.3038G>A	p.Arg1013Gln	hetero	10	AD/AR	VUS	
CDH23	NM_022124.5	c.1282G>A	p.Asp428Asn	hetero	10	AR/DR	VUS	
CDHR1	NM_001171971.2	c.601G>A	p.Glu201Lys	hetero	10	AR	VUS	
CDHR1	NM_001171971.2	c.700G>A	p.Val234Ile	hetero	10	AR	VUS	
CDHR1	NM_033100.3	c.2027T>A	p.Ile676Asn	homo	10	AR	VUS	
CEP290	NM_025114.3	c.1711+1G>A		hetero	12	AR	PV	
CEP290	NM_025114.3	c.14T>G	p.Ile5Arg	hetero	12	AR	VUS	
CHM	NM_000390.3	c.1718_1719del	p.Tyr573CysfsTer12	hemi	X		PV	
CHM	NM_000390	c.2T>A	p.Met1Lys	hemi	X	XL	VUS	

	.3							
CHM	NM_000390.3	c.652_655del	p.Ser218LysfsTer13	hemi	X	XL	LPV	
CHM	NM_000390.3	c.688delinsTG		hemi	X		LPV	
CNGA1	NM_001142564.1	c.398delG	p.Gly133fs	hetero	4	AR	PV	
CNGA1	NM_001142564.1	c.1067G>A	p.Arg356Lys	hetero	4	AD/AR	VUS	
CNGA1	NM_001142564.1	c.472del	p.Leu158fs	homo	4	AR	PV	
CNGA1	NM_001142564.1	c.2134C>T	p.Arg712Ter	hetero	4	AR	VUS	
CNGB1	NM_001297.4	c.217+5G>C		homo	16	AR	LPV	
CRB1	NM_001193640.1	c.1240C>T	p.Arg414Ter	hetero	1	AD/AR	PV	
CRB1	NM_201253.2	c.1576C>T	p.Arg526Ter	hetero	1	AD/AR	PV	
CRB1	NM_201253.2	c.2198A>G	p.Tyr733Cys	hetero	1	AD/AR	VUS	
CRB1	NM_001257965.1	c.550G>A	p.Gly184Arg		1	AD/AR	VUS	Novel
CRB1	NM_201253.2	c.653-2A>T		hetero	1	AD/AR	PV	
CYP4V2	NM_207352.3	c.1072G>T	p.Glu358Ter	homo	4	AR	LPV	
CYP4V2	NM_207352.3	c.219T>A	p.Phe73Leu	hetero	4	AR	VUS	
CYP4V2	NM_207352.3	c.675-1G>A		hetero	4	AR	LPV	Novel
CYP4V2	NM_207352.3	c.802_807A		hetero	4	AR	LPV	
CYP4V2	NM_207352.3	c.802-8_807del		hetero	4	AR	LPV	
CYP4V2	NM_207352.3	c.809_810C		hetero	4		LPV	
CYP4V2	NM_207352.3	c.992A>C	p.His331Pro	hetero	4		LPV	
EYS	NM_001142800.1	c.1382G>A	p.Cys461Tyr	hetero	6	AR	VUS	
EYS	NM_001142800.1	c.1963G>T	p.Gly655Ter	hetero	6	AR	PV	Novel
EYS	NM_001292009.1	c.1989del	p.Tyr664IlefsTer19	hetero	6	AR	LPV	Novel
EYS	NM_001142800.1	c.2259+1G>T	Splicing variant	hetero	6	AR	LPV	
EYS	NM_001142800.1	c.2528G>A	p.Gly843Glu	hetero	6	AR	LPV	
EYS	NM_001142800.1	c.2641+1G>A		hetero	6	AR	PV	Novel
EYS	NM_001142800.1	c.4957dup	p.Ser1653fs	hetero	6	AR	PV	
EYS	NM_001142800.1	c.4958delinsAG		hetero	6	AR	PV	Novel
EYS	NM_001292009.1	c.525_527del	p.Glu176del	hetero	6	AR	VUS	
EYS	NM_001142800.1	c.586A>C	p.Lys196Gln	hetero	6	AR	VUS	
EYS	NM_001142	c.6098_6105d	p.Gly2036Ilefs	hetero	6	AR	LPV	Novel

	800.1	up	Ter6					
EYS	NM_001292009.1	c.6117del	p.Val2040LeufsTer2	hetero	6	AR	LPV	Novel
EYS	NM_001142800.1	c.6557G>A	p.Gly2186Glu	hetero	6	AR	LPV	
EYS	NM_001142800.1	c.6571+6T>A		hetero	6	AR	VUS	Novel
EYS	NM_001142800.1	c.7394C>G	p.Thr2465Ser	hetero	6	AR	VUS	
EYS	NM_001142800.1	c.7492G>C	p.Ala2498Pro	hetero	6	AR	VUS	
EYS	NM_001142800.1	c.7679C>T	p.Pro2560Leu	hetero	1	AR	VUS	
EYS	NM_001142800.1	c.8805C>A	p.Tyr2935Ter	hetero	6	AR	PV	
EYS	NM_001292009.1	c.9095T>C	p.Ile3032Thr	hetero	6	AR	VUS	Novel
EYS	NM_001142800.1	c.9368delA	p.Asn3123fs	hetero	6	AR	LPV	
FAM161A	NM_001201543.1	c.1851-2A>T		homo	2	AR	LPV	Novel
FBN2	NM_001999.3	c.8279A>C	p.Glu2760Ala	hetero	5	AD	VUS	
FBN2	NM_001999.3	c.2633C>T	p.Ser878Leu	hetero	5	AD	VUS	
FSCN2	NM_001077182.2	c.1033C>T	p.Lue345Phe	hetero	17	AD	VUS	
GNAT1	NM_000172.3	c.814_817G		hetero	3	AD	VUS	
GNAT1	NM_000172.3	c.947A>G	p.Tyr316Cys	hetero	3	AD	VUS	
GNAT1	NM_144499.2	c.753 C>A	p.Asn251Lys	hetero	3		VUS	
HGSNAT	NM_152419.2	c.34_54del	p.Leu12_Leu18del	hetero	8	AR	VUS	Novel
HGSNAT	NM_152419.2	c.1030C>T	p.Arg344Cys	hetero	8	AR	VUS	
HK1	NM_033500.2	c.27+1G>C	splice_donor_variant	hetero	10	AD	LPV	
HK1	NM_001322365.1	c.83_84T		hetero	10	AD	LPV	Novel
IFT140	NM_014714.3	c.2137C>T	p.Arg713Trp	hetero	16	AR	VUS	
IFT140	NM_014714.3	c.217C>T	p.Arg73Trp	hetero	16	AR	VUS	
IFT140	NM_014714.3	c.1183G>A	p.Val395Met	hetero	16	AR	VUS	
IFT140	NM_014714.3	c.2551_2563del	p.Val851fs	hetero	16	AR	LPV	Novel
IMPDH1	NM_000883.3	c.947G>C	p.Arg316Pro	hetero	7	AD	LPV	Novel
IMPG1	NM_001282368.1	c.1586A>C	p.Gln529Pro		6		VUS	Novel
IMPG2	NM_016247.3	c.1589C>A	p.Ser530Ter	hetero	3	AD/AR	PV	
IMPG2	NM_016247.3	c.2629A>C	p.Met877Leu	hetero	3	AD/AR	VUS	
MAK	NM_001242957.2	c.493T>A	p.Tyr165Asn	hetero	6		VUS	
MAK	NM_001242	c.824C>A	p.Ala275Glu	hetero	6		VUS	Novel

	957.2							
MYO7A	NM_000260.3	c.1301G>A	p.Gly434Asp	hetero	11	AR	VUS	
MYO7A	NM_000260.3	c.2107G>A	p.Gly703Arg	hetero	11	AR	VUS	
MYO7A	NM_000260.3	c.488G>A	p.Gly163Glu	hetero	11	AR	VUS	
MYO7A	NM_000260.3	c.5930G>A	p.Arg1977Gln	hetero	11	AR	VUS	
NPHP1	NM_001128179.1	c.143G>A	p.Arg48Lys	hetero	2	AR	VUS	
NPHP1	NM_001128179.1	c.1578_1582G		hetero	2	AR	LPV	
NPHP4	NM_015102.4	c.1972C>T	p.Arg658Ter	hetero	1	AR	PV	
NPHP4	NM_015102.4	c.453-1G>C		hetero	1	AR	PV	Novel
NR2E3	NM_014249.3	c.646G>A	p.Gly216Ser	hetero	15	AD/AR	LPV	
NR2E3	NM_014249.3	c.355C>A	p.Gln119Lys	hetero	15	AD/AR	VUS	Novel
PCDH15	NM_001142763.1	c.1799+1G>T		hetero	10	AR/DR	PV	
PCDH15	NM_001142763.1	c.1795C>T	p.Arg599Ter	hetero	10	AR/DR	PV	
PDE6A	NM_000440.2	c.1957C>T	p.Arg653Ter	hetero	5	AR	PV	
PDE6A	NM_000440.2	c.2369G>A	p.Arg790His	hetero	5	AR	VUS	
PDE6B	NM_000283.3	c.1488del	p.Thr497fs	hetero	4	AR	PV	
PDE6B	NM_000283.3	c.1547T>C	p.Leu516Pro	hetero	4	AR	LPV	
PDE6B	NM_000283.3	c.1604T>A	p.Ile535Asn	homo	4	AR	LPV	
PDE6B	NM_000283.3	c.1669C>T	p.His557Tyr	homo	4	AR	LPV	
PDE6B	NM_000283.3	c.1712C>T	p.Thr571Met	hetero	4	AR	VUS	
PDE6B	NM_000283.3	c.2395C>T	p.Arg799Ter	hetero	4	AR	PV	
PDE6B	NM_000283.3	c.2492C>T	p.Ala831Val	hetero	4	AR	VUS	
PDE6B	NM_000283.3	c.592G>A	p.Gly198Ser	hetero	4	AD/AR	VUS	
PDE6B	NM_000283.3	c.712del	p.Val238fs	hetero	4	AR	LPV	Novel
PDE6B	NM_000283.3	c.815G>A	p.Arg272Gln	hetero	4	AD/AR	VUS	
PRCD	NM_001077620.2	c.2T>C	p.Met1?	homo	17	AR	PV	
PRPF31	NM_015629.3	c.1120C>T	p.Gln374Ter	hetero	19	AD	PV	
PRPF31	NM_015629.3	c.320T>C	p.Leu107Pro	hetero	19	AD	LPV	Novel
PRPF31	NM_015629.3	c.489delC	p.Ile164SerfsTer34	hetero	19	AD	LPV	Novel
PRPF31	NM_015629.3	c.1489delinsAT		hetero	19	AD	LPV	Novel
PRPF31	NM_015629	c.1060C>T	p.Arg354Ter	hetero	19	AD	PV	

	.3							
PRPF8	NM_006445.3	c.6902C>T	p.Pro2301Leu		17	AD	VUS	
PRPF8	NM_006445.3	c.6952_6953C		hetero	17	AD/AR	LPV	
PRPF8	NM_006445.3	c.1777C>T	p.Arg593Ter	hetero	17	AD	LPV	Novel
PRPH2	NM_000322.4	c.478C>T	p.Gln160Ter	hetero	6	AD/AR	PV	
RGR	NM_001012722.1	c.717_718G		hetero	10	AD/AR	VUS	
RHO	NM_000539.3	c.1040C>T	p.Pro347Leu	hetero	3	AD/AR	LPV	
RHO	NM_000539.3	c.310G>A	p.Val104Ile	hetero	3	AD/AR	VUS	
RHO	NM_000539.3	c.36del	p.Phe13fs	hetero	3	AD/AR	PV	
RHO	NM_000539.3	c.403C>T	p.Arg135Trp	hetero	3	AD/AR	LPV	
RHO	NM_000539.3	c.50C>T	p.Thr17Met	hetero	3	AD/AR	LPV	
RHO	NM_000539.3	c.965_966delinsAA	p.Cys322Ter	hetero	3	AD/AR	LPV	Novel
RHO	NM_000539.3	c.994G>C	p.Glu332Gln	hetero	3	AD/AR	VUS	Novel
RP1	NM_006269.1	c.2238_2239del	p.Ser747Ter	hetero	8	AD/AR	LPV	Novel
RP1	NM_006269.1	c.2296C>T	p.Gln766Ter	hetero	8	ADAR	PV	
RP1	NM_006269.1	c.256C>A	p.Pro86Thr	hetero	8	AD/AR	VUS	
RP1	NM_006269.1	c.4196del	p.Cys1399fs	hetero	8	AD/AR	PV	
RP1	NM_006269.1	c.4196delG	p.Cys1399LeufsTer5	hetero	4	AD/AR	PV	
RP1	NM_006269.1	c.5797C>T	p.Arg1933Ter	hetero	8	AD/AR	PV	
RP1	NM_006269.1	c.5913C>A	p.Asn1971Lys	hetero	8	ADAR	VUS	
RP1	NM_006269.1	c.5971C>T	p.Gln1991Ter	hetero	8	AD/AR	LPV	
RP1	NM_006269.1	c.6178_6179G		hetero	8	AD/AR	LPV	
RP1	NM_006269.1	c.6179delinsGA		hetero	8	AD/AR	LPV	Novel
RP1	NM_006269.1	c.6181del	p.Ile2061fs	hetero	8	AD/AR	VUS	
RP1	NM_006269.1	c.6353G>A	p.Ser2118Asn	hetero	8	AD/AR	VUS	
RP1L1	NM_178857.5	c.347C>T	p.Pro116Leu	hetero	8	AD	VUS	Novel
RP1L1	NM_178857.5	c.2123C>A	p.Ser708Ter	hetero	8		LPV	
RP2	NM_006915.2	c.353G>A	p.Arg118His	hemi	X	XL	LPV	
RP9	NM_203288.1	c.380A>G	p.Asn127Ser	hetero	7	AD	VUS	Novel
RPGR	NM_001034853.1	c.2032G>T	p.Glu678Ter	hemi	X	XL	LPV	
RPGR	NM_001034	c.2405_2406d	p.Glu802fs	hemi	X	XL	PV	

	853.1	el						
RPGR	NM_000328.2	c.1599delinsTGACG		hemi	X	XL	LPV	
RS1	NM_000330.3	c.78+1G>A		hemi	X		LPV	
SEMA4A	NM_001193300.1	c.1120C>A	p.Pro374Thr	hetero	1	AD/AR	VUS	
SNRNP200	NM_014014.4	c.1202A>G	p.Glu401Gly	hetero	2	AD	VUS	Novel
SNRNP200	NM_014014.4	c.2041C>T	p.Arg681Cys	hetero	2	AD	LPV	
TGFBI	NM_000358.2	c.371G>A	p.Arg124His	hetero	5		PV	
TULP1	NM_001289395.1	c.800T>G	p.Met267Arg	hetero	6	AR	VUS	Novel
TULP1	NM_001289395.1	c.986T>C	p.Phe329Ser	hetero	6	AR	PV	
USH1G	NM_173477.4	c.164+5G>A		homo	17	AR	VUS	
USH2A	NM_206933.2	c.10931C>T	p.Thr3644Met	hetero	1	AR	VUS	
USH2A	NM_206933.2	c.11136_11137del	p.Gln3714fs	hetero	1	AR	LPV	Novel
USH2A	NM_206933.2	c.11156G>A	p.Arg3719His	hetero	1	AR	PV	
USH2A	NM_007123.5	c.1143G>A	p.Gln381Gln	hetero	1	AR	LPV	
USH2A	NM_007123.5	c.1184C>T	p.Thr395Met	hetero	1	AR	VUS	
USH2A	NM_206933.2	c.13339A>G	p.Met4447Val	hetero	1	AR	VUS	
USH2A	NM_206933.2	c.1450C>T	p.Gln484Ter	hetero	1	AR	PV	Novel
USH2A	NM_206933.2	c.14557A>G	p.Met4853Val	hetero	1	AR	VUS	
USH2A	NM_206933.2	c.15178T>C	p.Ser5060Pro	hetero	1	AR	VUS	
USH2A	NM_206933.2	c.15518T>C	p.Leu5173Pro	hetero	1	AR	VUS	Novel
USH2A	NM_206933.2	c.202C>T	p.His68Tyr	hetero	1	AR	VUS	
USH2A	NM_206933.2	c.2802T>G	p.Cys934Tr	hetero	1	AR	LPV	
USH2A	NM_007123.5	c.4070C>T	p.Thr1357Met	hetero	1	AR	VUS	
USH2A	NM_206933.2	c.451G>C	p.Ala151Pro	hetero	1	AR	VUS	Novel
USH2A	NM_206933.2	c.6326-1G>T		hetero	1	AR	PV	
USH2A	NM_206933.2	c.7046G>A	p.Trp2349Ter	hetero	1	AR	PV	Novel
USH2A	NM_206933.2	c.7880T>C	p.12627Thr	hetero	1	AR	VUS	Novel
USH2A	NM_206933.2	c.8254G>A	p.Gly2752Arg	hetero	1	AR	VUS	
USH2A	NM_206933.2	c.8559-2A>G		hetero	1	AR	PV	
USH2A	NM_206933.2	c.9258+1G>T		hetero	1	AR	PV	
VPS13B	NM_017890	c.11468G>C	p.Gly3823Ala	hetero	8	AR	VUS	Novel

	.4							
VPS13B	NM_017890 .4	c.7220_7221 A		hetero	8	AR	PV	
WDR19	NM_025132 .3	c.1613G>T	p.Gly538Val	hetero		AR	VUS	Novel
WDR19	NM_025132 .3	c.2645+1G>T		hetero		AR	LPV	

Abbreviations: HGVS, Human Genome Variation Society; ACMG, American College of Medical Genetics and Genomics; AD, autosomal dominant; AR, autosomal recessive; XL, x-linked inheritance; PV, pathogenic variant; LPV, likely pathogenic variant; VUS, variant of unknown significance

Figure S1. Ocular characteristics of patients with unexpected genetic results. From left to right: wide fundus photographs, fundus autofluorescence images, optical coherence tomography images, and electroretinogram results from patients with unexpected genotypes. **a)** A woman in her mid-20s with retinal pigmentary dystrophy from pseudoxanthoma elasticum due to *ABCC6*. **b)** A teenage boy with reticular pigmentary dispersions in choroideremia caused by *CHM*. **c)** A woman in her early-50s showing chorioretinal degeneration with yellow-white crystals in Bietti crystalline dystrophy associated with *CYP4V2*. **d)** A man in his mid-40s showing peripheral retinal degeneration with foveal retinoschisis caused by *RS1*. **e)** A teenage boy with corneal dystrophy concomitant with retinal pigmentary degeneration due to *TGFB1*. **f)** A woman in her early-40s with Cohen syndrome; retinal dystrophy and mental impairment caused by *VPS13B*. **g)** A woman in her early-20s with retinal pigmentary change associated with Senior-Løken syndrome due to *WDR19*.

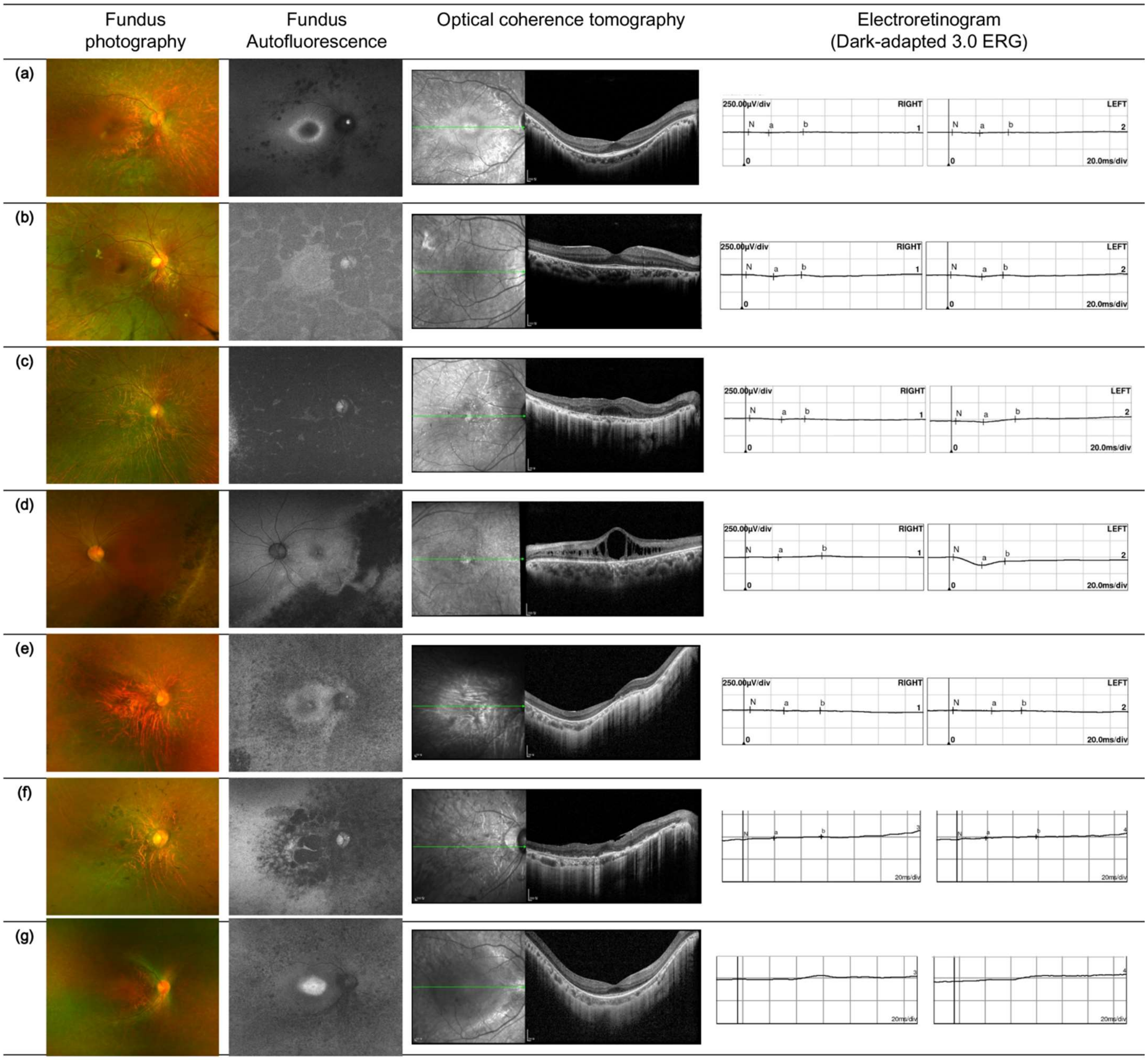

Table S5. List of patients with inconclusive results.

Genetic results										Phenotype suggested by genotype	Reason for inconclusive results
**Subject No.**	**Causative gene**	**HGVS DNA change**	**HGVS protein change**	**Zygosity**	**Inheritance**	**Origin**	**ACMG criteria**				
30-40	SNRNP200	c.3268C>T	p.R1090W	Hetero	AD	Unknown	VUS	PM2,PM5,PP2,PP3		RP	○ Wild type mother and carrier healthy sister<br>○ Impossible to determine whether there was a difference of penetrance between sister and the patient or another genetic cause
41-60	GNAT1	c.814_817G		Hetero	AD	Unknown	VUS	PM2,PM4	Novel	CSNB	○ Affected father from history taking, wild type healthy sister and carrier son without symptoms<br>○ Ophthalmologic findings including pigmentary fundus, CME, and extinguished ERG not compatible with CSNB from AD inherited *PDE6B*
	GNAT1	c.947A>G	p.Y316C	Hetero	AD	Unknown	VUS	PM2,PP2,PP3			
48-71	IMPG1	c.1586A>C	p.Q529P	Hetero	AD	Paternal	VUS	PM2	Novel	Vitelliform macular dystrophy	○ *IMPG1* in the patient and her affected brother from affected father<br>○ Fundus findings including localized pigmentation around supero-temporal main arcade with CME not compatible with vitelliform dystrophy
59-94	CEP290	c.1711+1G>A		Hetero	AR	Unknown	PV	PVS1,PM2,PP5		Joubert syndrome	○ No family history of RP<br>○ Physical examination showed no evidence of systemic manifestation except retinal
	CEP290	c.14T>G	p.Ile5Arg	Hetero	AR	Unknow	VUS	PM2,PM5,	Novel		

						n		BP1			change
71-109	GNAT1	c.753 C>A	p.Asn251Lys	Hetero	AD	Paternal	VUS	PP2, PP3		CSNB	○ *GNAT1* from asymptomatic father<br>○ Ophthalmologic findings including diffuse pigmentary changes and low amplitude of ERG response not compatible with CSNB
175-227	CAPN5	c.976A>T	p.T326S	Hetero	AD	De novo	VUS	PS2,PM2,PP2,BP4	Novel	Vitreoretinopathy, neovascular inflammatory	○ No family history of RP and true de-novo *CAPN5* variant<br>○ Fundus findings of diffuse pigmentation and bullseye pattern autofluorescence do not match the phenotypes of neovascular inflammatory vitreoretinopathy
216-294	CRB1	c.1240C>T	p.R414X	Hetero	AD	Unknown	PV	PVS1,PM2, PP5		PPCA	○ Her two sons revealed one with wild type and one as an asymptomatic carrier<br>○ Extensive pigmentary atrophy of the entire retinal tissue with spared macula is controversial to conclude with PPCA

Abbreviations: HGVS, Human Genome Variation Society; ACMG, American College of Medical Genetics and Genomics; AD, autosomal dominant; AR, autosomal recessive; PV, pathogenic variant; VUS, variant of unknown significance; BP, benign supporting; PM, pathogenic moderate; PP, pathogenic supporting; PS, pathogenic strong; PVS, pathogenic very strong; PPCA, pigmented paravenous chorioretinal atrophy; CSNB, congenital stationary night blindness; CME, cystoid macular edema; RP, retinitis pigmentosa

Figure S2. Clinical characteristics of patients with inconclusive results. From left to right: wide fundus photographs and fundus autofluorescence images from both eyes, spectral-domain optical coherence tomography, Goldmann kinetic visual field test, and electroretinogram results examined from the same eye of patients with unexpected genotypes.

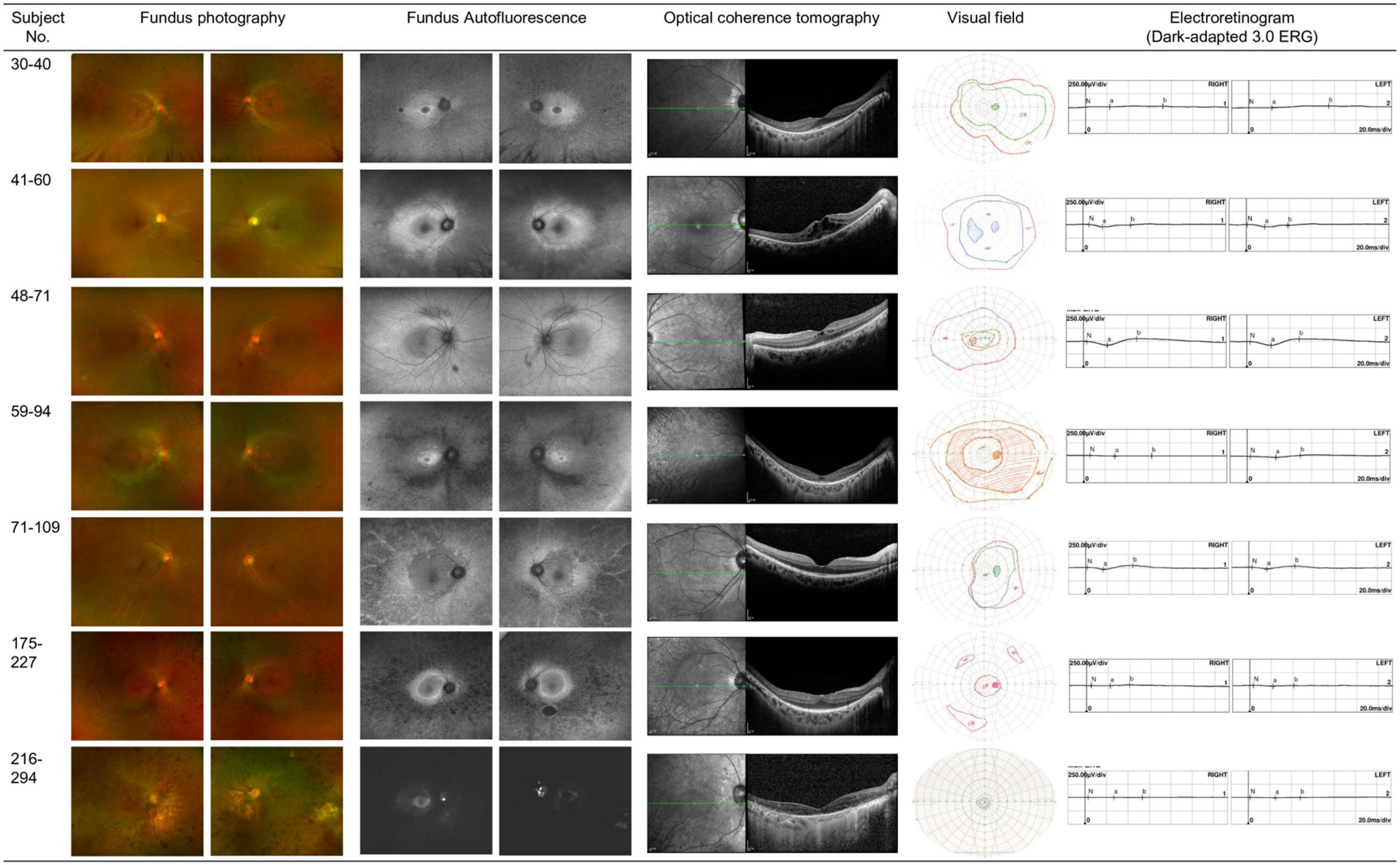

Supplement: Supplementary file 1 [file genes-12-00675-s001.zip › genes-1171954-supplementary.pdf]
